# Quantitative flow ratio or angiography for the assessment of non-culprit lesions in acute coronary syndromes, a randomized trial

**DOI:** 10.1007/s00392-024-02484-5

**Published:** 2024-07-09

**Authors:** Helen Ullrich-Daub, Maximilian Olschewski, Boris Schnorbus, Khelifa-Anis Belhadj, Till Köhler, Markus Vosseler, Thomas Münzel, Tommaso Gori

**Affiliations:** 1 Department of Cardiology, Cardiology I, University Medical Center Mainz, Langenbeckstrasse 1, 55131 Mainz, Germany; 2 German Centre for Cardiovascular Research (DZHK), Standort RheinMain, Frankfurt, Germany; 3 Cardiopraxis Mainz and Ingelheim, Mainz, Germany

**Keywords:** Acute coronary syndrome, Percutaneous coronary interventions, Quantitative flow ratio, Angiography

## Abstract

**Background:**

Patients undergoing percutaneous coronary intervention for acute coronary syndromes often have multivessel disease (MVD). Quantitative flow ratio (QFR) is an angiography-based technology that may help quantify the functional significance of non-culprit lesions, with the advantage that measurements are possible also once the patient is discharged from the catheterization laboratory.

**Aim:**

Our two-center, randomized superiority trial aimed to test whether QFR, as compared to angiography, modifies the rate of non-culprit lesion interventions (primary functional endpoint) and improves the outcomes of patients with acute coronary syndromes and MVD (primary clinical endpoint).

**Methods:**

In total, 202 consecutive patients (64 [56–71] years of age, 160 men) with STEMI (*n* = 69 (34%)), NSTEMI (*n* = 94 (47%)), or unstable angina (*n* = 39 (19%)) and MVD who had undergone successful treatment of all culprit lesions were randomized 1:1 to angiography- vs. QFR-guided delayed revascularization of 246 non-culprit stenoses (1.2/patient).

**Results:**

The proportion of patients assigned to percutaneous intervention was not different between groups (angiography group: 45 (45%) vs. QFR: 56 (55%), *P* = 0.125; relative risk = 0.80 (0.60–1.06)). At 12 months, a primary clinical endpoint event (composite of death, nonfatal myocardial infarction, revascularization, and significant angina) occurred in 24 patients (angiography-guided) and 23 patients (QFR-guided; *P* = 0.637, HR = 1.16 [0.63–2.15]). None of its components was different between groups.

**Discussion:**

QFR guidance based on analysis of images from the primary intervention was not associated with a difference in the rate of non-culprit lesion staged revascularization nor in the 12-month incidence of clinical events in patients with acute coronary syndromes and multivessel disease.

**Trial registration number:**

ClinicalTrials.gov Registry (NCT04808310).

**Graphical abstract:**

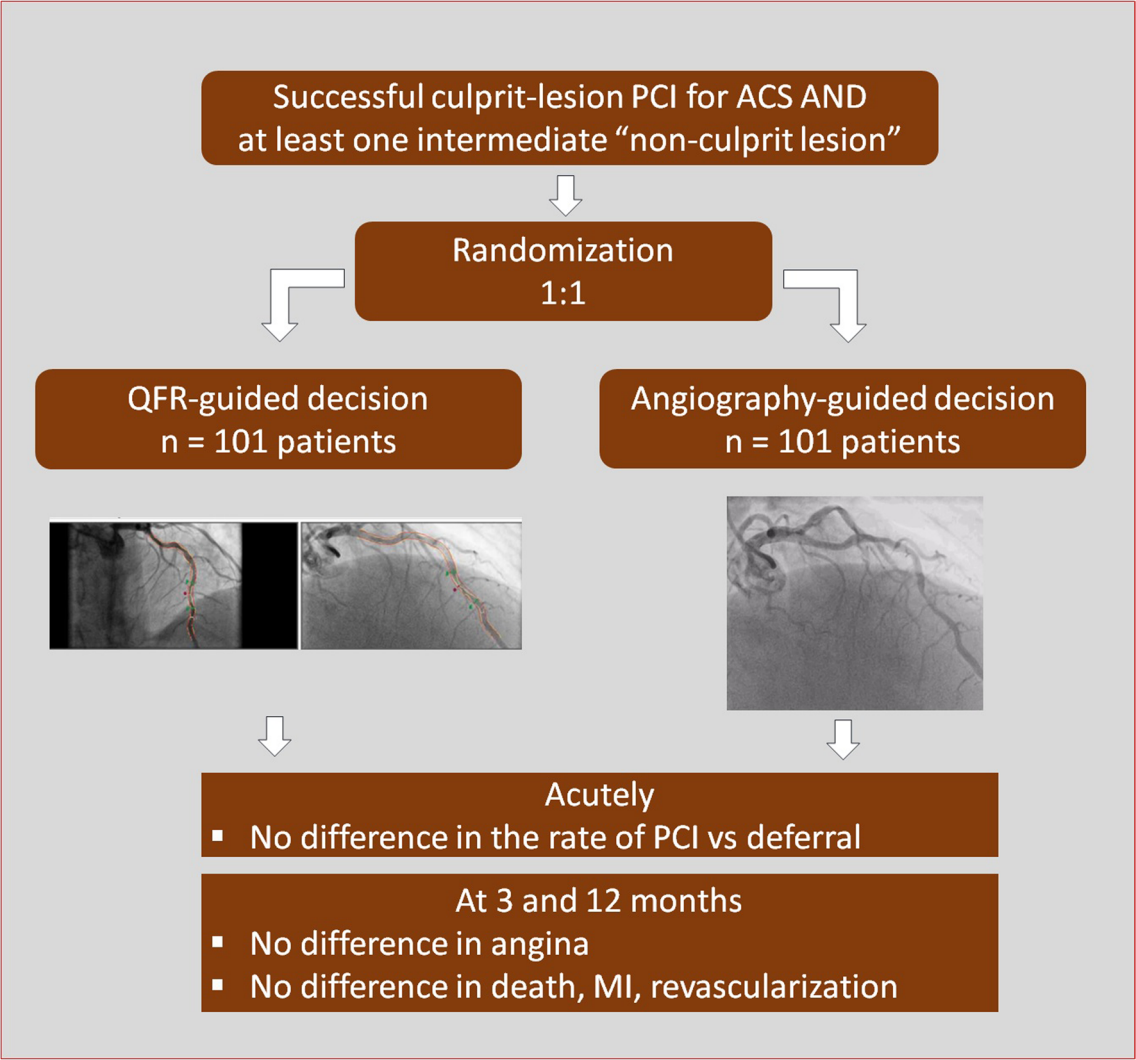

## Introduction

Approximately 50% of the patients undergoing percutaneous coronary intervention (PCI) for an acute coronary syndrome (ACS) have additional non-culprit lesions [1]. Any decision regarding the treatment of these lesions can be based on visual estimation or on the assessment of their functional significance. While wire-based assessment of fractional flow reserve consistently results in a decrease in the proportion of patients undergoing PCI versus deferral and might also yield improved clinical outcomes [2–4], this method remains rarely used in clinical routine in ACS. Beyond potential considerations related to the stability of the boundary conditions that need to be satisfied for the assessment of fractional flow reserve [5], one practical limitation to the widespread use of wire-based technologies is that any assessment must be performed at the time of primary PCI or in a separate invasive exam. Quantitative flow ratio (QFR) provides an analysis based on (wire-free) three-dimensional quantitative angiography which can be theoretically applied to images taken at the time of primary PCI. In the context of non-culprit lesions in ACS patients, QFR has been validated in terms of feasibility, accuracy when compared to wire-based assessment, reproducibility, and correlation with the myocardial area at risk [6–11]. Based on this background, we hypothesized that, similar to fractional flow reserve, QFR might result in a reduction in the number of patients referred for staged PCI of non-culprit lesions and, possibly, might improve outcomes with the advantage of allowing post hoc, off-line analysis.

## Materials and methods

### Study design

In a two-center, blinded, parallel, superiority trial, ACS patients with MVD (at least one additional 30–90% diameter stenosis as assessed by the operator and confirmed by quantitative coronary analysis) were randomized to angiography-guided or QFR-guided assessment of non-culprit lesions after successful PCI of all culprit lesions. The protocol (NCT04808310), published in Ullrich et al. [12], was approved by the local ethics committee (2020–15296). All patients gave informed consent.

Key exclusion criteria were persistent symptoms or evidence of ischemia after PCI of the culprit lesion(s); stenoses or patients not amenable to treatment with PCI; settings in which QFR is not approved (previous coronary artery bypass graft surgery; chronic total occlusion; heart failure; severe pulmonary or valvular disease; atrial fibrillation; bifurcation or ostial lesions); and non-culprit lesions with residual QFR < 0.80.

First, QFR was measured in all vessels with non-culprit lesions in both groups to confirm residual QFR > 0.80 (residual QFR is the QFR calculated in the hypothetical absence of the target lesion. A QFR < 0.80 assumes that treatment of this/these focal lesion(s) would not remove the source of ischemia, such as in the case of diffuse disease). These QFR measurements were performed by certified staff not involved in the treatment of the patients with QAngioXA3D (Medis, Leiden, Netherlands). Then, randomization was performed in a 1:1 ratio with random blocks. In the angiography arm, decisions regarding the treatment of non-culprit lesions were based on angiography only (consensus of two expert interventionalists blinded to QFR results). In the QFR arm, physicians were instructed to treat all lesions (one or more per vessel) with a QFR < 0.80. QFR analysis was performed using contrast flow velocity.

**Fig. 1:**
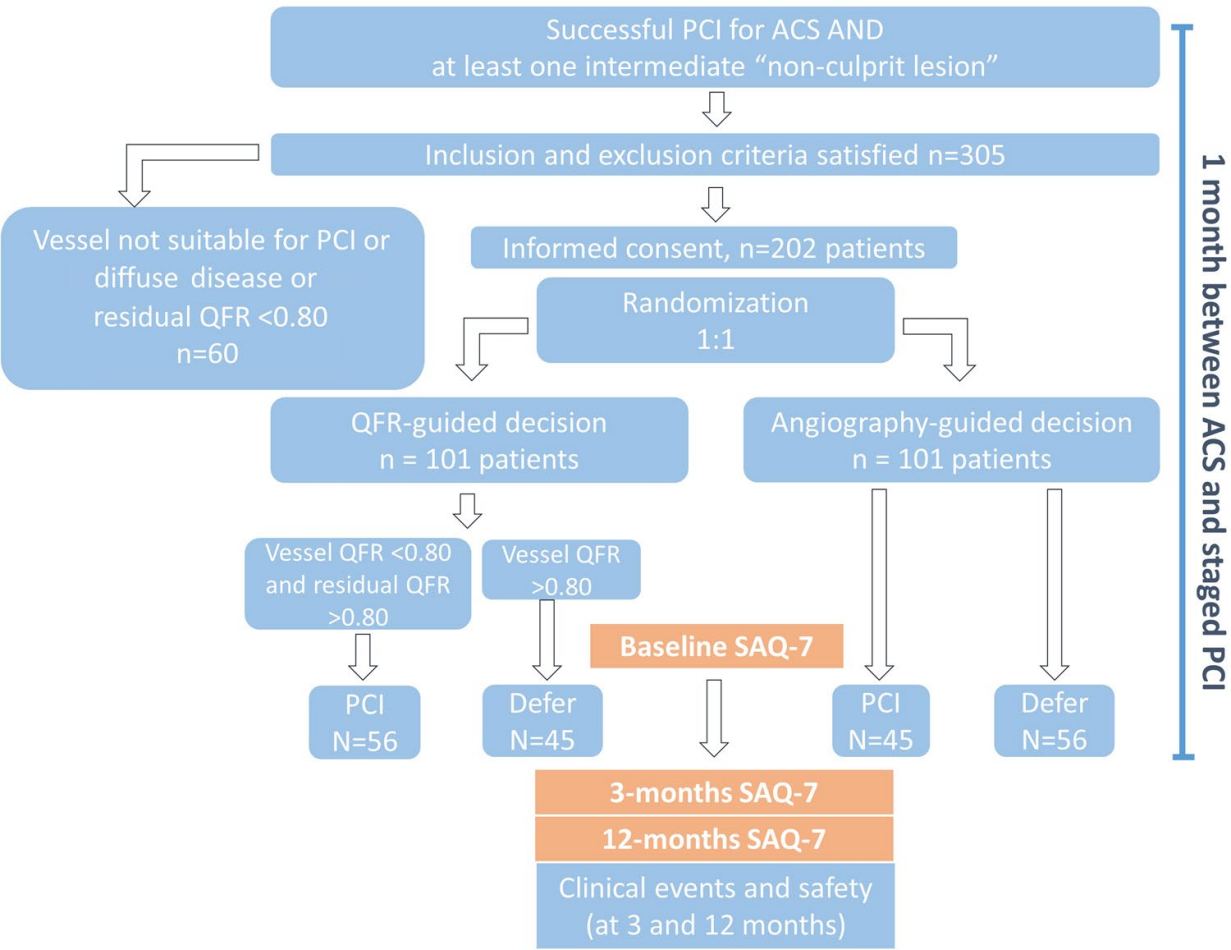
Protocol, patient workflow (upper box), and decision tree. A total of 202 patients with acute coronary syndrome and multivessel disease were randomized 1:1 to an angiography-guided or quantitative flow ratio (QFR) strategy for treating non-culprit lesions

Staged non-culprit PCIs were performed in both groups 4 weeks after ACS. Participants were blinded to the group allocation; unblinded use of QFR or wire-based indexes at this time was not allowed. Figure 1 presents the flow diagram of the study.

### Endpoints

Angina (assessed using the SAQ-7 questionnaire) and clinical follow-up data were collected at 3 and 12 months after protocol-mandated complete revascularization. Adherence to the protocol and all critical data were monitored. The primary endpoint (functional) was the proportion of patients assigned to medical treatment in the two groups (QFR vs. angiography). The primary endpoint (clinical) was a composite of all-cause death, non-fatal myocardial infarction (including periprocedural type 4a myocardial infarction), unplanned hospitalization for angina or heart failure, unplanned revascularization, and SAQ < 90 at 12 months. Secondary endpoints are listed in the protocol paper [12]. An external blinded committee adjudicated clinical events.

### Statistics

Statistical analysis was performed with MedCalc (Ostend, Belgium) on the intention to treat population. Data are presented as number (percentage) or means (± SD). The physiological endpoint was tested as the difference in proportions and relative risk between groups estimated with exact 95% confidence intervals and *P*-values. The proportion of patients with the clinical endpoint (composite of all-cause death, non-fatal myocardial infarction, target vessel revascularization, unplanned hospitalization for angina, significant angina at the Seattle Angina Questionnaire) within 12 months was analyzed using Kaplan–Meier plots; treatment effects were estimated with Cox models and presented as hazard ratios (95% confidence intervals). Subgroup analyses of the primary end point were performed according to target vessel (left anterior descending artery or non-left anterior descending artery), initial presentation (MI or non-MI), sex (male or female), age (< 65 years or ≥ 65 years), diabetes mellitus, left ventricular ejection fraction (≤ 55% or > 55%), and type of P2Y12 inhibitors (clopidogrel, ticagrelor, or prasugrel). Missing values were not replaced or imputed. Sample size calculations for the functional endpoint (% of patients in whom PCI was deferred) were based on the results of the FLOWER-MI trial [13] in which PCI was performed in 388 patients (66.2%) in the FFR-guided group and 560 patients (97.1%) in the angiography-guided group. Conservatively, we hypothesized that as many as 13% of the patients in the angiography-guided group would be deferred (in the FAMOUS-NSTEMI [14], in which lesions up to 90% were included, sample size calculations were based on a projected deferral rate of 15%). In the QFR group, we hypothesized that the deferral rate would be 33% (similar to the rate observed in the FLOWER-MI and the FORZA studies [13, 15]). Based on this hypothesis, 176 patients would be required to have a 90% chance of detecting, as significant at the 5% level, a difference in the primary outcome measure from 33% in the QFR group to 13% in the angiography group [12]. For the clinical endpoint, an incidence of 14.8% at 1 year was reported in the FFR group of the FORZA trial [15], while in the angiography group of the FAME trial it was 32.4% [16]. A sample size of 176 would yield a power of 80% at a significance level < 5%.

## Results

Patient characteristics are presented in Table 1. From October 2020 to June 2022, 202 patients (64 [56–71] years of age, 160 men) with STEMI (*n* = 69 (34%)), NSTEMI (*n* = 94 (47%)), or unstable angina (*n* = 39 (19%)) and multivessel disease were enrolled and underwent randomization to angiography-guided or QFR-guided treatment of non-culprit lesions (*n* = 101 each). Two hundred and thirty-five culprit lesions were treated with drug-eluting stents before enrollment with procedural success rates of 100% (inclusion criteria). Thirty-three patients had more than one culprit lesion. Twenty-four patients had atrial fibrillation and received dual therapy with novel anticoagulant and clopidogrel (aspirin until discharge). All other ones received potent P2Y12 inhibitors.

**Table 1:** Patient characteristics

	Angiography (*n* = 101)	QFR (*n* = 101)
Clinical characteristics		
Age, years	66 ± 11	61 ± 10
Male sex, %	76 (76%)	84 (83%)
BMI, kg/m^2^	28 ± 4	28 ± 4
Hyperlipidemia, %	70 (70%)	71 (71%)
Hypertension, %	72 (71%)	73 (73%)
Smoking, %	28 (28%)	28 (28%)
Prior smoking, %	20 (20%)	14 (14%)
Family history of CAD, %	20 (20%)	28 (28%)
Previous PCI, %	20 (20%)	14 (14%)
Previous CABG, %	1 (1%)	0 (0%)
Previous stroke, %	5 (5%)	6 (6%)
COPD, %	6 (6%)	0 (0%)
LVEF, %	53 ± 8	53 ± 7
Creatinine, mg/dl	1.0 ± 0.6	1.0 ± 0.3
Atrial fibrillation, %	8(8%)	16(16%)
Clinical presentation		
Unstable angina, %	15 (15%)	24 (24%)
NSTEMI, %	50 (50%)	44 (44%)
STEMI, %	36 (36%)	33 (33%)
Culprit lesion LAD, %	47 (47%)	44 (44%)
Culprit lesion LCX, %	31 (31%)	35 (35%)
Culprit lesion RCA, %	37 (37%)	35 (35%)
Peak troponin, p/ml	26575 ± 41495	19660 ± 39790
Peak troponin > 10 × URL, %	81(80%)	71(70%)
Therapy at discharge		
Aspirin, %	89 (88%)	93 (92%)
Clopidogrel, %	23 (23%)	11 (11%)
Prasugrel, %	60 (60%)	72 (71%)
Ticagrelor, %	17 (17%)	16 (16%)
B-blocker, %	74 (73%)	71 (70%)
Statins, %	97 (96%)	95 (94%)
Angiographic and QFR findings		
Patients with non-culprit LAD, %	46 (46%)	47 (47%)
Non-culprit LCX, %	37 (37%)	30 (30%)
Non-culprit RCA, %	40 (40%)	46 (46%)
Patients with intermediate lesion(s) in 1 vessel, %	79 (78%)	79 (78%)
Patients with intermediate lesion(s) in 2 vessels, %	22 (22%)	22 (22%)
Mean ± SD angiographic severity in %	54 ± 14	55 ± 15
Angiographic severity > 50%	59 (59%)	65 (65%)
Angiographic severity > 70%	15 (15%)	18 (18%)
Mean QFR	-	0.79 ± 0.16
Lesions with QFR < 0.80, LAD, %	-	23 (49%)
Lesions with QFR < 0.80, LCX, %	-	13 (43%)
Lesions with QFR < 0.80, RCA, %	-	22 (48%)

*BMI* body mass index, *CAD* coronary artery disease, *PCI* percutaneous coronary intervention, *CABG* coronary artery bypass graft, *COPD* chronic obstructive pulmonary disease, *LVEF* left ventricular ejection fraction, *NSTEMI* non-ST elevation myocardial infarction, *LAD* left anterior descending, *LCX* left circumflex, *RCA* right coronary artery

### Primary functional endpoint and staged PCI

**Fig. 2:**
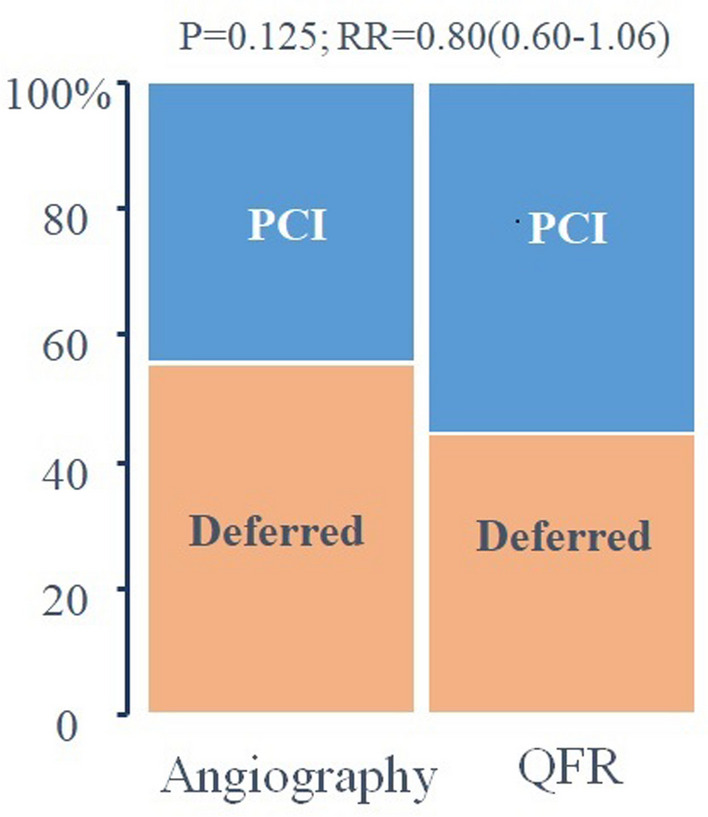
Hemodynamic endpoint. There was no difference between groups in the rate of referral to PCI

The angiographic severity of the lesions was similar between groups (Table 1). In the QFR group, mean non-culprit lesion was 0.79 ± 0.16; 58/124 lesions (47%) showed a QFR < 0.80. The proportion of patients referred to PCI for non-culprit lesions was similar in the two groups (45 (45%) in the angiography vs. 56 (55%) in the QFR group, *P* = 0.125; relative risk 0.80 (0.60–1.06), Fig. 2). This observation did not change when only MI patients were included (45% vs 51%, *P* = 0.549). There were no events between primary PCI and staged non-culprit PCIs, which were performed as per protocol at 4 weeks using newer generation drug-eluting stents. Use of advanced diagnostics (imaging or physiology) was prohibited at that time. There were two protocol deviations (one patient in each group with indication to PCI was treated conservatively). The technical success rate was 100% in patients who underwent staged PCI. The number of stents implanted (angiography: 1.6 ± 1.1; QFR: 1.4 ± 0.7, *P* = 0.445) and their length (angiography: 26.9 ± 20.2 mm; QFR: 25.8 ± 14.6 mm, *P* = 0.756) were similar between groups. Periprocedural troponin elevation > 5 times the 99th percentile (without other signs of ischemia) was observed in 13 patients following the staged procedures (six in the QFR group, *P* = 0.704). No patient showed troponin elevations > 35 times the 99th percentile.

### Follow-up

**Fig. 3:**
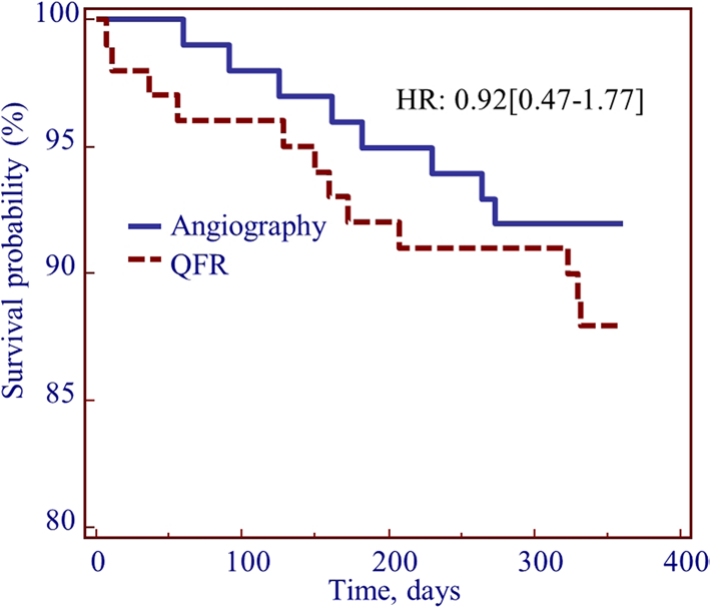
The use of QFR did not have an impact on the incidence of the composite endpoint (death, myocardial infarction, stroke, hospitalization for angina or heart failure, significant angina) at 12-month follow-up

Two patients in each group were lost to a 12-month follow-up; data were complete for all others. Up to 12 months, there were two deaths (one in each group, *P* = 0.480), four unplanned PCIs (two each, *P* = 0.615), one stroke (in the QFR group, *P* = 0.992), six bleedings requiring medical attention (four in the QFR group, *P* = 0.674), 15 hospitalizations (eight in the QFR group, *P* = 0.998), and 22 patients (11 in each group, *P* = 0.822) had an SAQ < 90. The incidence of the combined endpoint (angiography group: 18 events, QFR: 19 events, *P* = 0.855, relative risk = 1.06 [0.59–1.89], Fig. 3) was not different between groups. This observation did not change in several subgroup analyses (Table 2). Neither minimum lumen diameter nor reference vessel diameter was associated with the primary endpoint (MLD: 1.00 [0.93–1.07]; RVD: 0.98 [0.92–1.05]) nor did they influence the relationship between group and incidence of events. Only age was a predictor of events (*P* = 0.015) in multivariate analysis. The mean SAQ score at 3 and 12 months was also not different between groups (Table 3).

**Table 2:** Subgroup analysis of the primary outcome

Subgroup	Angiography group	QFR group	HR(CI)
	Number of events (%)		
Primary presentation			
MI	14 (16)	15 (19)	1.22 (0.59–2.51)
Unstable angina	4 (27)	3 (12.5)	0.52 (0.12–2.30)
STEMI vs NSTEMI vs UA			0.82 (0.52–1.28
Sex			
Male	10 (13)	16 (18)	1.40 (0.64–3.08)
Female	8 (32)	2 (20)	0.61 (0.13–2.87)
Culprit lesion LAD			
Yes	10 (21)	9 (20)	1.00 (0.41–2.45)
No	8 (15)	9 (16)	1.09 (0.42–2–82)
EF			
> 55%	2 (14)	2 (12)	0.92 (0.13–6.50)
< 55%	15 (18)	14 (17)	0.98 (0.47–2.02)
Type of P2Y12			
Clopidogrel	2 (9)	1 (9)	1.05 (0.10–11.47)
Prasugrel	10 (17)	12 (17)	1.03 (0.45–2.47)
Ticagrelor	6 (35)	5 (31)	1.02 (031–3.32)
Use of B-blockers	17 (24%)	17 (23%)	0.83 (0.42–1.66)

**Table 3:** SAQ scores at baseline (4 weeks after ACS), and 3 and 12 months

	Group			
	Angiography		QFR	
	Mean	SD	Mean	SD
SAQ 7 score—baseline	94	14	94	14
SAQ 7 score—3 months	94	14	94	15
SAQ 7 score—12 months	96	11	95	11
SAQ angina frequency—baseline	6.0	0.2	6.0	0.1
SAQ angina frequency—3 months	6.0	0.3	5.9	0.6
SAQ angina frequency—12 months	6.0	0.0	5.9	0.5
SAQ angina stability—baseline	5.4	1.2	5.6	1.1
SAQ angina stability—3 months	5.6	1.1	5.5	1.1
SAQ angina stability—12 months	5.8	0.7	5.7	0.8
SAQ Physical limitation—baseline	4.8	0.5	4.8	0.6
SAQ Physical limitation—3 months	4.9	0.5	4.8	0.6
SAQ Physical limitation—12 months	4.9	0.5	4.9	0.3
SAQ quality of life—baseline	4.7	0.8	4.7	0.9
SAQ quality of life—3 months	4.7	0.9	4.7	0.9
SAQ quality of life—12 months	4.8	0.8	4.8	0.8
SAQ Treatment Satisfaction—baseline	4.7	0.8	4.7	0.8
SAQ Treatment Satisfaction—3 months	4.6	0.9	4.7	0.9
SAQ Treatment Satisfaction—12 months	4.7	0.8	4.8	0.8

## Discussion

Multivessel disease occurs in 40 to 65% of patients with acute myocardial infarction [1]. As a consequence, angina despite successful PCI of culprit lesions is frequent and incomplete revascularization has been associated with an increased risk of adverse clinical outcomes [1, 17, 18]. In an effort to correctly identify potentially ischemia-inducing non-culprit lesions, a number of studies investigated whether hemodynamic assessment might provide benefits. Wire-based technologies have consistently shown to reduce the number of non-culprit lesions (and patients) referred to PCI [13, 14, 19], and in the FAMOUS-NSTEMI, FRAME-MI, and FIRE trials, use of physiology-based assessment was also associated with an improved prognosis [2–4]. Despite this evidence, a number of reasons limit the use of physiology-based assessments in the setting of ACS. These include cost concerns and device availability, (perceived) lack of conclusive evidence, and a discussion regarding the stability of the boundary conditions (recruitability of the microcirculation) in ACS. More practically, considerations that limit the use of these methods in daily routine include the unwillingness/impossibility to prolong procedural times in the acute setting and detection of additional intermediate lesions only upon revision of the angiogram. QFR might represent an alternative to the gold standard (wire-based) fractional flow reserve with the theoretical advantage of not requiring additional catheterization laboratory time in the acute setting and allowing post hoc analysis after the primary PCI is completed and the patient is discharged from the catheterization laboratory [8, 11]. A recent large trial in an all-comer setting showed that QFR leads to a change in the clinical decision in about 20% of the patients, followed by improved outcomes as shown by a reduction in myocardial infarction and unplanned PCI [20]. In the ACS setting, QFR has been shown provide reproducible and accurate results [6–11], but its impact on deferral rate and outcomes has not been definitely demonstrated. Our randomized trial tested the hypothesis that QFR, as compared to angiography, would reduce the rate of patients referred for non-culprit PCI while also improving their 1-year outcomes.

Regarding the physiological endpoint, the expected reduction in the rate of patients referred to PCI for non-culprit lesions was not observed in the current trial. Of note, the proportion of patients assigned to medical therapy in our QFR group was in line with that reported in the fractional flow reserve group in previous trials [4, 13]; in contrast, this proportion was higher in our angiography-guided group as compared to what was observed in previous studies.

Similar considerations apply to the clinical endpoint. In the current study, the incidence of death, myocardial infarction, and unplanned revascularization was similar in both groups and comparable to that seen in the fractional flow reserve group of FRAME-MI and FLOWER-MI. The rate of events was also lower than that observed in the angiography group in the FLOWER-MI [4, 13]. These observations appear to suggest that a more conservative approach (lower rate of non-culprit PCIs, whether QFR- or angiography-guided), as applied in both patients groups in the current study, might improve outcomes in ACS patients. This notion appears to be confirmed by a recent post hoc analysis of FRAME-MI in which the incidence of follow-up events was lowest in patients who did not receive staged PCI as compared to those who underwent both appropriate (QFR < 0.80) and inappropriate (QFR > 0.80) PCI [21].

### Limitations

Although powered for endpoints used in FORZA and FAME, this was a small trial. Notably, patients with non-focal non-culprit lesions (residual QFR < 0.80) were not enrolled to reduce this potential source of confounders, thus allowing a smaller sample size.

To test the use of QFR in clinical practice, we analyzed a broad range of lesions (30–90% by QCA), including the whole spectrum in which fractional flow reserve is known to lead to a reclassification of stenosis severity and a consequent improvement in 1-year outcomes [2, 22]. A very similar range was used in multiple other studies testing similar endpoints [10, 15, 23].

In order to compare QFR with routine practice, we did not introduce a wire-based assessment arm. This comparison would have been interesting.

Our conclusions contrast with those of FAVOR III [24]. Importantly, QFR requires adherence of a specific protocol of image acquisition. In previous trials, angiography was performed after randomization and images were acquired according to these requirements in the QFR group. In clinical routine of ACS, however, focus is set on the treatment of culprit lesions; often, any discussion on the treatment of intermediate lesions is undertaken after the patient’s discharge from the catheterization laboratory and is based on standard-quality images. To reflect clinical practice in the setting of ACS, our study tested the hypothesis that QFR may offer an advantage over angiography based on an analysis of standard images not acquired for the specific purpose of QFR.

Finally, patients with STEMI, NSTEMI, and unstable angina were enrolled in this study. Patients with unstable angina were less than 20% of the total, and sensitivity analyses excluding this group did not yield different results.

### Perspectives

In this two-center, blinded, randomized study with external adjudication of events, QFR does not appear superior to angiography in guiding complete revascularization in multivessel disease patients with ACS.

## Abbreviations

ACS: Acute coronary syndrome
NSTEMI: Non-ST-segment elevation myocardial infarction
PCI: Percutaneous coronary intervention
QFR: Quantitative flow ratio
SAQ: Seattle Angina Questionnaire
STEMI: ST-segment elevation myocardial infarction
TIMI: Thrombolysis in Myocardial Infarction (Study Group)

## References

[1] Montone RA, Niccoli G, Crea F, Jang IK (2020) Management of non-culprit coronary plaques in patients with acute coronary syndrome. Eur Heart J 41(37):3579–3586 32676644 10.1093/eurheartj/ehaa481

[2] Layland J, Oldroyd KG, Curzen N et al (2015) Fractional flow reserve vs. angiography in guiding management to optimize outcomes in non-ST-segment elevation myocardial infarction: the British Heart Foundation FAMOUS-NSTEMI randomized trial. Eur Heart J 36(2):100–111 25179764 10.1093/eurheartj/ehu338 PMC4291317

[3] Biscaglia S, Guiducci V, Escaned J et al (2023) Complete or culprit-only PCI in older patients with myocardial infarction. N Engl J Med 389(10):889–898 37634150 10.1056/NEJMoa2300468

[4] Lee JM, Kim HK, Park KH et al (2023) Fractional flow reserve versus angiography-guided strategy in acute myocardial infarction with multivessel disease: a randomized trial. Eur Heart J 44(6):473–484 36540034 10.1093/eurheartj/ehac763

[5] De Bruyne B, Adjedj J (2015) Fractional flow reserve in acute coronary syndromes. Eur Heart J 36(2):75–76 25179765 10.1093/eurheartj/ehu362

[6] Xing Z, Pei J, Huang J, Hu X, Gao S (2019) Diagnostic performance of QFR for the evaluation of intermediate coronary artery stenosis confirmed by fractional flow reserve. Braz J Cardiovasc Surg 34(2):165–172 30916126 10.21470/1678-9741-2018-0234 PMC6436789

[7] Dettori R, Frick M, Burgmaier K et al (2021) Quantitative flow ratio is associated with extent and severity of ischemia in non-culprit lesions of patients with myocardial infarction. J Clin Med 10(19):4535 34640551 10.3390/jcm10194535 PMC8509261

[8] Erbay A, Penzel L, Abdelwahed YS et al (2021) Feasibility and diagnostic reliability of quantitative flow ratio in the assessment of non-culprit lesions in acute coronary syndrome. Int J Cardiovasc Imaging 37(6):1815–1823 33651231 10.1007/s10554-021-02195-2 PMC8255265

[9] Lauri FM, Macaya F, Mejia-Renteria H et al (2020) Angiography-derived functional assessment of non-culprit coronary stenoses in primary percutaneous coronary intervention. EuroIntervention 15(18):e1594–e1601 31543501 10.4244/EIJ-D-18-01165

[10] Milzi A, Dettori R, Marx N, Reith S, Burgmaier M (2021) Quantitative flow ratio (QFR) identifies functional relevance of non-culprit lesions in coronary angiographies of patients with acute myocardial infarction. Clin Res Cardiol 110(10):1659–1667 34251507 10.1007/s00392-021-01897-w PMC8484103

[11] Tebaldi M, Biscaglia S, Erriquez A et al (2021) Comparison of quantitative flow ratio, Pd/Pa and diastolic hyperemia-free ratio versus fractional flow reserve in non-culprit lesion of patients with non ST-segment elevation myocardial infarction. Catheter Cardiovasc Interv Catheter Cardiovasc Interv. 98(6):1057–1065 33211381 10.1002/ccd.29380

[12] Ullrich H, Olschewski M, Belhadj KA, Munzel T, Gori T (2022) Quantitative flow ratio or angiography for the assessment of non-culprit lesions in acute coronary syndromes: protocol of the randomized trial QUOMODO. Front Cardiovasc Med 9:815434 35445090 10.3389/fcvm.2022.815434 PMC9013799

[13] Puymirat E, Cayla G, Simon T et al (2021) Multivessel PCI guided by FFR or angiography for myocardial infarction. N Engl J Med 385(4):297–308 33999545 10.1056/NEJMoa2104650

[14] Berry C, Layland J, Sood A et al (2013) Fractional flow reserve versus angiography in guiding management to optimize outcomes in non-ST-elevation myocardial infarction (FAMOUS-NSTEMI): rationale and design of a randomized controlled clinical trial. Am Heart J 166(4):662-668 e3 24093845 10.1016/j.ahj.2013.07.011 PMC3807653

[15] Burzotta F, Leone AM, Aurigemma C et al (2020) Fractional flow reserve or optical coherence tomography to guide management of angiographically intermediate coronary stenosis: a single-center trial. JACC Cardiovasc Interv 13(1):49–58 31918942 10.1016/j.jcin.2019.09.034

[16] Tonino PA, Fearon WF, De Bruyne B et al (2010) Angiographic versus functional severity of coronary artery stenoses in the FAME study fractional flow reserve versus angiography in multivessel evaluation. J Am Coll Cardiol 55(25):2816–2821 20579537 10.1016/j.jacc.2009.11.096

[17] Faro DC, Laudani C, Agnello FG et al (2023) Complete percutaneous coronary revascularization in acute coronary syndromes with multivessel coronary disease. JACC: Cardiovasc Interv 16(19):2347–64 37821180 10.1016/j.jcin.2023.07.043

[18] Bainey KR, Engstrom T, Smits PC et al (2020) Complete vs culprit-lesion-only revascularization for ST-segment elevation myocardial infarction: a systematic review and meta-analysis. JAMA Cardiol 5(8):881–888 32432651 10.1001/jamacardio.2020.1251 PMC7240651

[19] Smits PC, Abdel-Wahab M, Neumann FJ et al (2017) Fractional flow reserve-guided multivessel angioplasty in myocardial infarction. N Engl J Med 376(13):1234–1244 28317428 10.1056/NEJMoa1701067

[20] Xu B, Tu S, Song L et al (2021) Angiographic quantitative flow ratio-guided coronary intervention (FAVOR III China): a multicentre, randomised, sham-controlled trial. Lancet 398(10317):2149–2159 34742368 10.1016/S0140-6736(21)02248-0

[21] Lee SH, Hong D, Shin D et al (2023) QFR assessment and prognosis after nonculprit PCI in patients with acute myocardial infarction. JACC: Cardiovasc Interv 16(19):2365–2379 37821181 10.1016/j.jcin.2023.08.032

[22] Baptista SB, Raposo L, Santos L et al (2016) Impact of routine fractional flow reserve evaluation during coronary angiography on management strategy and clinical outcome: one-year results of the POST-IT. Circ Cardiovasc Interv 9(7):e003288 27412867 10.1161/CIRCINTERVENTIONS.115.003288

[23] Song L, Tu S, Sun Z et al (2020) Quantitative flow ratio-guided strategy versus angiography-guided strategy for percutaneous coronary intervention: rationale and design of the FAVOR III China trial. Am Heart J 223:72–80 32179258 10.1016/j.ahj.2020.02.015

[24] Song L, Xu B, Tu S et al (2022) 2-year outcomes of angiographic quantitative flow ratio-guided coronary interventions. J Am Coll Cardiol 80(22):2089–2101 36424680 10.1016/j.jacc.2022.09.007

