# Publisher Correction: Quantitative flow ratio or angiography for the assessment of non-culprit lesions in acute coronary syndromes, a randomized trial

**DOI:** 10.1007/s00392-024-02497-0

**Published:** 2024-07-29

**Authors:** Helen Ullrich-Daub, Maximilian Olschewski, Boris Schnorbus, Khelifa-Anis Belhadj, Till Köhler, Markus Vosseler, Thomas Münzel, Tommaso Gori

**Affiliations:** 1 Department of Cardiology, Cardiology I, University Medical Center Mainz, Langenbeckstrasse 1, 55131 Mainz, Germany; 2 German Centre for Cardiovascular Research (DZHK), Standort RheinMain, Frankfurt, Germany; 3 Cardiopraxis Mainz und Ingelheim, Mainz, Germany

**Publisher Correction: Clinical Research in Cardiology**

10.1007/s00392-024-02484-5

When this article was published, previous author correction had been accidentally lost during a second proof run. Most of these concerned textual changes. Seriously affected were the full name of the 4th author who appeared as "Anis Belhadj" though it should be correctly "Khelifa-Anis Belhadj" and the first sentence in the Results section of the Abstract. Instead of "The proportion of patients assigned to medical treatment versus percutaneous intervention …" it should start correctly "The proportion of patients assigned to percutaneous intervention …".

The Original article has been corrected.

The Publisher apologises for this mistake.

